# Loss of ATRX Does Not Confer Susceptibility to Osteoarthritis

**DOI:** 10.1371/journal.pone.0085526

**Published:** 2013-12-30

**Authors:** Lauren A. Solomon, Bailey A. Russell, David Makar, Nathalie G. Bérubé, Frank Beier

**Affiliations:** 1 Departments of Paediatrics and Biochemistry, Schulich School of Medicine & Dentistry, The University of Western Ontario, London, Ontario, Canada; 2 Children’s Health Research Institute, London, Ontario, Canada; 3 Department of Physiology and Pharmacology, Schulich School of Medicine & Dentistry, The University of Western Ontario, London, Ontario, Canada; INSERM U606 - university Paris 7, France

## Abstract

The chromatin remodelling protein ATRX is associated with the rare genetic disorder ATR-X syndrome. This syndrome includes developmental delay, cognitive impairment, and a variety of skeletal deformities. ATRX plays a role in several basic chromatin-mediated cellular events including DNA replication, telomere stability, gene transcription, and chromosome congression and cohesion during cell division. We have used a loss-of-function approach to directly investigate the role of Atrx in the adult skeleton in three different models of selective Atrx loss. We specifically targeted deletion of *Atrx* to the forelimb mesenchyme, to cartilage and to bone-forming osteoblasts. We previously demonstrated that loss of ATRX in forelimb mesenchyme causes brachydactyly while deletion in chondrocytes had minimal effects during development. We now show that targeted deletion of *Atrx* in osteoblasts causes minor dwarfism but does not recapitulate most of the skeletal phenotypes seen in ATR-X syndrome patients. In adult mice from all three models, we find that joints lacking Atrx are not more susceptible to osteoarthritis, as determined by OARSI scoring and immunohistochemistry. These results indicate that while ATRX plays limited roles during early stages of skeletal development, deficiency of the protein in adult tissues does not confer susceptibility to osteoarthritis.

## Introduction

 Osteoarthritis (OA) is a degenerative joint disease to which there is no cure. It is characterized by the degeneration of articular cartilage and changes in other joint tissues including subchondral bone and synovium. Cartilage is maintained by a balance of both anabolic and catabolic activities. OA occurs when these processes are in disequilibrium and catabolism outweighs anabolic repair[1]. Osteoarthritis can be triggered by many factors including diet, injury, strain and genetic abnormalities[1–5]. However, the molecular mechanisms driving disease onset and progression are incompletely understood.

 Alterations in epigenetic mechanisms affecting gene expression have been previously reported in articular chondrocytes[4]. A recent study has linked genetic variants of *DOT1L*, an evolutionarily conserved histone methyltransferase required for chondrogenic differentiation, to increased susceptibility to osteoarthritis[6]. Alterations in expression of histone acetylases and deacetylases have also been associated with arthritis[7]. The aging-related gene *SirT1* is of particular interest, as it codes for a protein capable of deacetylating histones and other proteins[8]. Expression of SirT1 in chondrocytes is associated with increased survival and down-regulation of the proapoptotic protein PTP1B associated with OA[9]. Age-related diseases, as well as normal aging, are frequently influenced by changes in chromatin structure, leading to deleterious effects on cell and tissue function[10]. 

 Hypomorphic mutations causing dysfunction of the ATRX chromatin remodeling protein can lead to various skeletal deformities, including dwarfism, spine deformities and malformation of the hands and feet[11,12]. These defects occur in conjunction with developmental delay, psychomotor and mental retardation, distinct facial features, urogenital abnormalities and α-thalassemia[11]. Radiological analysis in a few cases has shown that individuals with *ATRX* mutations show delayed bone age[11]. ATRX contains two conserved domains where the majority of disease-related mutations are located. The N-terminus contains a plant homeodomain-type (PHD-type) zinc finger that acts as a histone reader module by mediating binding to specific post-translational modifications on histone H3[13–15]. Towards the C-terminus, ATRX contains a Sucrose Non Fermenting 2 (SNF2)-type DNA-dependent ATPase domain that functions to remodel chromatin[16–18]. The functional full-length protein has been shown to play an important role in chromosomal integrity, maintaining organization and sister chromatid cohesion during cell division[19].

While there have been various developmental studies that show severe effects of tissue-specific Atrx deficiency[20–23] cartilage-specific inactivation of the Atrx gene in mice does not result in major phenotypes[24]. On the other hand, loss of Atrx in the forebrain and pituitary of mice leads to features of aging[25], prompting us to ask whether Atrx is important in aging skeletal elements and joints. Since ATRX is highly expressed in chondrocytes[24], it could be a potential epigenetic regulator of specific genes involved in healthy cartilage maintenance and its loss may lead to diseases such as osteoarthritis. This study examined the role of ATRX in three models of Atrx deficiency and assessed the onset and progression of osteoarthritis using Osteoarthritis Research Society International (OARSI) histopathology guidelines and molecular markers of OA. 

## Materials and Methods

### Animals

The cartilage-specific Atrx depleted *Atrx* ^fl/yCol2a1cre+^ (Atrx^Col2^) mice were generated as described previously[24]. Both wild-type and Atrx^Col2^ mice were maintained until the age of two years. Bone-specific Atrx depleted mice were generated using an osteoblast-specific cre mouse[26] to generate *Atrx* ^*fl/yCol1a1cre+*^ (Atrx^Col1^) mice. Forelimb specific deletion of Atrx was generated as described previously using the forelimb-specific *Prx1-cre* [27,28]. Since Atrx is X-linked, male mice resulting from these crosses carry one copy of the *Atrx* gene containing the loxP sites and Cre-positive males were conditionally *Atrx*-null. All animals in this study were male mice from the first generation of this cross between B57Bl/6 and 129/SV backgrounds. This study was conducted in strict accordance with Canadian Council on Animal Care (CCAC) guidelines for ethical use and care of animals. All experiments involving animals were approved by the Council on Animal Care at the University of Western Ontario (Permit number: 2007-045-10).

PCR genotyping from ear tissue for Cre and Atrx as described previously[24]. *Atrx* ^*floxed*^ and *Cre* alleles were confirmed as previously described [29,30].. A 1.5 kb fragment containing the neo cassette within the *Atrx* floxed allele was identified with one set of primers (5′-GATCGGCCATTGAACAAGAT-3′ and 5′-ATA GGT CGG CGG TTC AT-3′) whereas the wild-type allele was detected with another set (5′-CCC GAG TAT CTG GAA GAC AG-3′and 5′-ATA GGT CGG CGG TTC AT-3′). Primers (5′-CCT GGA AAA TGC TTC TGT CC-3′) and (5′-CAG GGT GTT ATA AAC AAT CCC-3′) were used to amplify a common 300 base pair fragment for all three versions of the *cre* gene. PCR conditions were as follows: 95°C for 3 min (95°C for 30 s, 55°C for 45 s, and 72°C for 1 min) × 36, 72°C for 10 min for Cre , 95°C for 3 min (95°C for 30 s, 55°C for 1 min, and 72°C for 5 min) × 36, 72°C for 10 min for Atrx..

### Histological staining of bone and joint sections

Limbs were dissected from Atrx-depleted mice upon sacrifice and processed for histological analyses by Picrosirius red, safranin-O and immunohistochemical staining as described[31–33]. For histological analyses, specimens were fixed in 4% paraformaldehyde, decalcified with 5% EDTA, paraffin embedded and sectioned at 5μm. For immunohistochemistry, samples were examined in dewaxed paraffin–embedded sections. Following dehydration, sections were blocked in hydrogen peroxide followed by 5% goat serum, and incubated with a polyclonal rabbit antibodies to MMP13 (Santa Cruz), aggrecan fragments (MMP Cleaved, N-terminus FFGVG neoepitope, Millipore), Atrx (Prestige Antibodies, Sigma-Aldrich) or type II collagen (Santa Cruz) according to manufacturer's instructions. Following overnight incubation at 4°C sections were incubated with HRP–conjugated secondary goat antibody against rabbit IgG and detected by staining with DAB (Dako). To quantify trabecular area, sections were stained for one hour in 0.1% Picrosirius Red. Using imageJ software, an area of interest (AOI) was set from the chondro-osseous junction to 200μm below the growth plate in the trabecular bone area of the mineralized zone, as described previously[25]. 

### Histological end-stage analysis

Sections from a minimum of three independent litters were stained with Safranin-O and fast green, and graded according to the Osteoarthritis Research Society International (OARSI) scoring system[34]. OA scores were assigned by two blinded observers and total score was calculated by averaging the grade and stage values for each slide (a minimum score of 0 represents no OA degradation and a score of 6 represents the maximum degree of OA). Maximal OARSI grade was determined for each joint based on the surface displaying the highest OARSI score in each section. 

### Skeletal stains and measurements

Live mice were weighed at P0 and P21. Animals were skinned and eviscerated, fixed overnight in 95% ethanol followed by overnight fixation in acetone for Alizarin Red/Alcian Blue staining. Whole skeletons were stained for 7–10 days (0.05% Alizarin Red, 0.015% Alcian Blue, 5% acetic acid in 70% ethanol)[31]. Skeletons were cleared in 2% w/w KOH. Bones were imaged on an Olympus SP-57OUZ camera. Limb bones and skulls from at least three independent littermate pairs were measured using a dissecting microscope with a ruler.

### Microcomputed tomography

Male *Atrx* ^*Col1*^ and control littermate mice were sacrificed at weaning (21 days of age), skinned and eviscerated, followed by fixation in formalin. Whole bodies were imaged using a scanner (eXplore Locus MicroCT; GE Healthcare) at 120 kV and 20 mA, with a 0.154 mm3 voxel resolution with a total of 900 slices per scan. Bone mineral density (BMD), cortical thickness and trabecular numbers were calculated using the Microview 3D visualization and analysis software (MicroView, Version 2.1.2, GE Healthcare Biosciences)[35].

### Osteoblast isolation and differentiation

Calvarial cells were isolated from 8-10 day old *ATRX* ^*Col1*^ mice using sequential collagenase digestion, as previously described[36,37]. Briefly, calvaria from control and mutant mice were dissected, scraped clean of mesenchyme by micro dissection, and digested through three changes of collagenase. Pooled cells from the latter two changes of collagenase were plated on six-well plates at a density of 1.5 × 10^4^ cells/cm^2^ in culture medium. After three days, cells were transferred to high density micromass cultures in 24-well plates, with each well containing 5 × 10^4^ cells in a 150-μl drop of culture medium. After 12 hours, culture medium was added to the micromass cultures. Culture media was supplemented with 50 μg/ml ascorbic acid and 2 mM β-glycerophosphate, and cultures were maintained for up to 4 weeks.

### Analyses of alkaline phosphatase activity and mineral deposition

Calvarial micromass cultures were fixed in 4% PFA overnight at 4°C. Alkaline phosphatase activity was assayed using a mixture of 1.25 mM Fast Red B salt in 0.1 M Tris-HCl, pH 8, and  0.25 mM naphthol AS-MX phosphate, protected from light for one hour. Mineralised nodules were visualised following ALP staining using  2.5% silver nitrate solution for 1 h with exposure to light.

## Results

### Skeletal phenotypes of adult *Atrx* ^*Col2*^ mice

 To determine if Atrx played a direct role in skeletal development, mice with cartilage-specific inactivation of Atrx were generated utilizing the Cre-LoxP system. Female mice previously engineered with *LoxP* sites flanking exon 18 of *Atrx* [20]. were crossed with male mice expressing Cre recombinase under the control of the mouse collagen II (*Col2a1*) promoter[30]. Cre-positive male offspring lacked Atrx in the developing cartilage but developed normally with only minor defects in skeletogenesis[24]. *Atrx* ^*Col2*^ mice were born at normal Mendelian ratios and did not demonstrate defects in skeletal size or growth plate morphology at birth or weaning[24].

**Figure 1:**
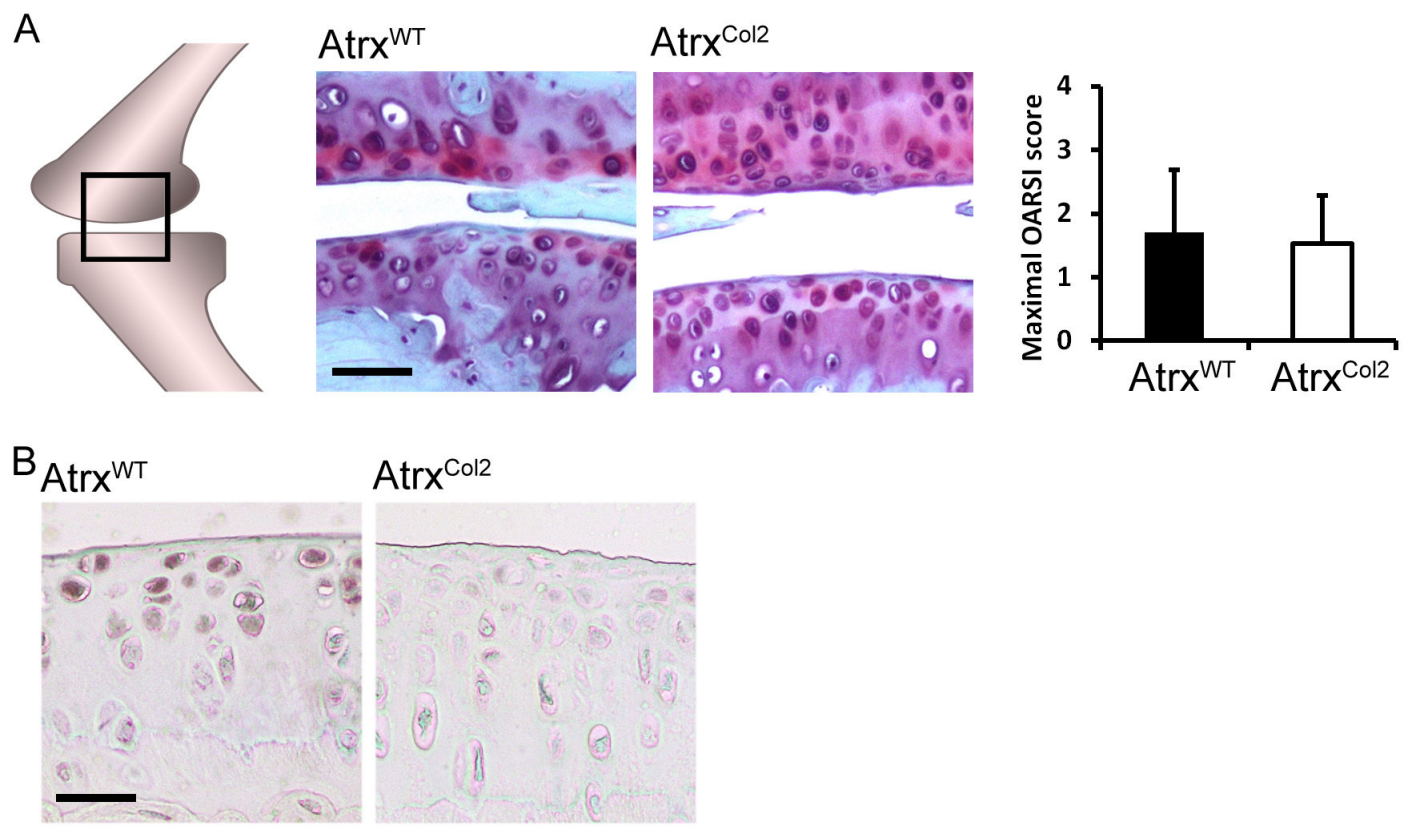
Characterisation of joint morphology in two year-old *Atrx^Col2^* mice. A) Minor fibrillation and fissuring of the tibial and femoral articular surfaces was observed in both genotypes, demonstrating mild osteoarthritis in both genotypes. OARSI scoring confirmed the absence of significant differences between genotypes. Scale bar = 96 µm. B) Immunohistological staining of articular cartilage, demonstrating that ATRX protein is present in normal aged articular cartilage, but absent from the nuclei of mutant chondrocytes. Scale bar = 48 µm.

 To examine if embryonic depletion of *Atrx* in the developing cartilage contributes to reduced cartilage integrity later in life, two-year old *Atrx* ^*Col2*^ mice were examined for signs of osteoarthritis and other skeletal defects. No significant weight difference was observed between control and mutant mice at two years of age (Ctrl: 36.57 ± 6.38 grams, *Atrx* ^*Col2*^: 31.56 ± 8.51 grams). Sections of knee joints from control and mutant *Atrx* ^*Col2*^ mice were assessed for osteoarthritis using the OARSI histopathology guidelines[34] on tissue sections stained for cartilage proteoglycan with safranin-O. In both genotypes, cartilage appeared relatively healthy with only minor degeneration. Maximal OARSI score of the knee joint was not different in controls compared to *Atrx* ^*Col2*^ mice. Control mice had a maximal knee score 1.70 and mutant mice had a maximal score of 1.53 (p= 0.89) (Figure 1A). *Atrx* ^*Col2*^ mice did not show a significant increase in  fibrillation and fissuring of the articular surfaces. Absence of ATRX protein in articular cartilage was confirmed by immunohistochemistry using a rabbit anti-ATRX antibody (Prestige Antibodies, Sigma-Aldrich) on sections from knee (Figure 1B) and elbow joints (not shown). 

### MMP13, Aggrecan fragment and Collagen 2 levels are normal in *Atrx^Col2^* mice

 Several molecular changes have been associated with OA. MMP13 (matrix metalloproteinase 13) plays a catabolic role in the progression of cartilage degeneration and cleaves collagens, gelatin, and aggrecan[3,38]. Aggrecan is an extracellular matrix protein and its degradation into fragments is accelerated in OA[39]. Finally, type II collagen is cleaved by a number of MMPs including MMP13. In arthritic joints, loss of the collagen matrix is accelerated, leading to erosion of the articular surface[34]. 

 Using immunohistochemistry, we detected no visible difference in Mmp13 levels in tibia articular cartilage between control and mutant mice. The level of Mmp13 in the cartilage was quantified as a percentage of Mmp13 positive cells to total cells in 1000 pixel wide sections of articular cartilage. Statistical analysis confirmed that there was no significant difference in Mmp13 levels between mutants and control littermates (35.7% vs. 39.3% respectively, p= 0.451) (Figure S1A,B). Tibial aggrecan fragment levels was also not altered between control and *Atrx* ^*Col2*^ mice as revealed by IHC analysis. Control and knockout mice were not significantly different in the percentage of aggrecan fragment-positive cells versus total cells in articular cartilage(34.02% vs. 22.87%, p= 0.074) (Figure S1C, D). Upon immunostaining for type II collagen, we observed that the mean thickness of the type II collagen -positive zone of the articular cartilage was not significantly different when comparing the tibiae of control and mutant mice (87.993 μm vs. 90.065 μm, p= 0.837). There was also no significant difference in the thickness of type II collagen-positive articular cartilage between the femurs of controls and mutant mice (82.080 μm vs. 81.142 μm, p= 0.923)(Figure S1E,F).

### Loss of ATRX in during forelimb development does not lead to forelimb osteoarthritis

**Figure 2:**
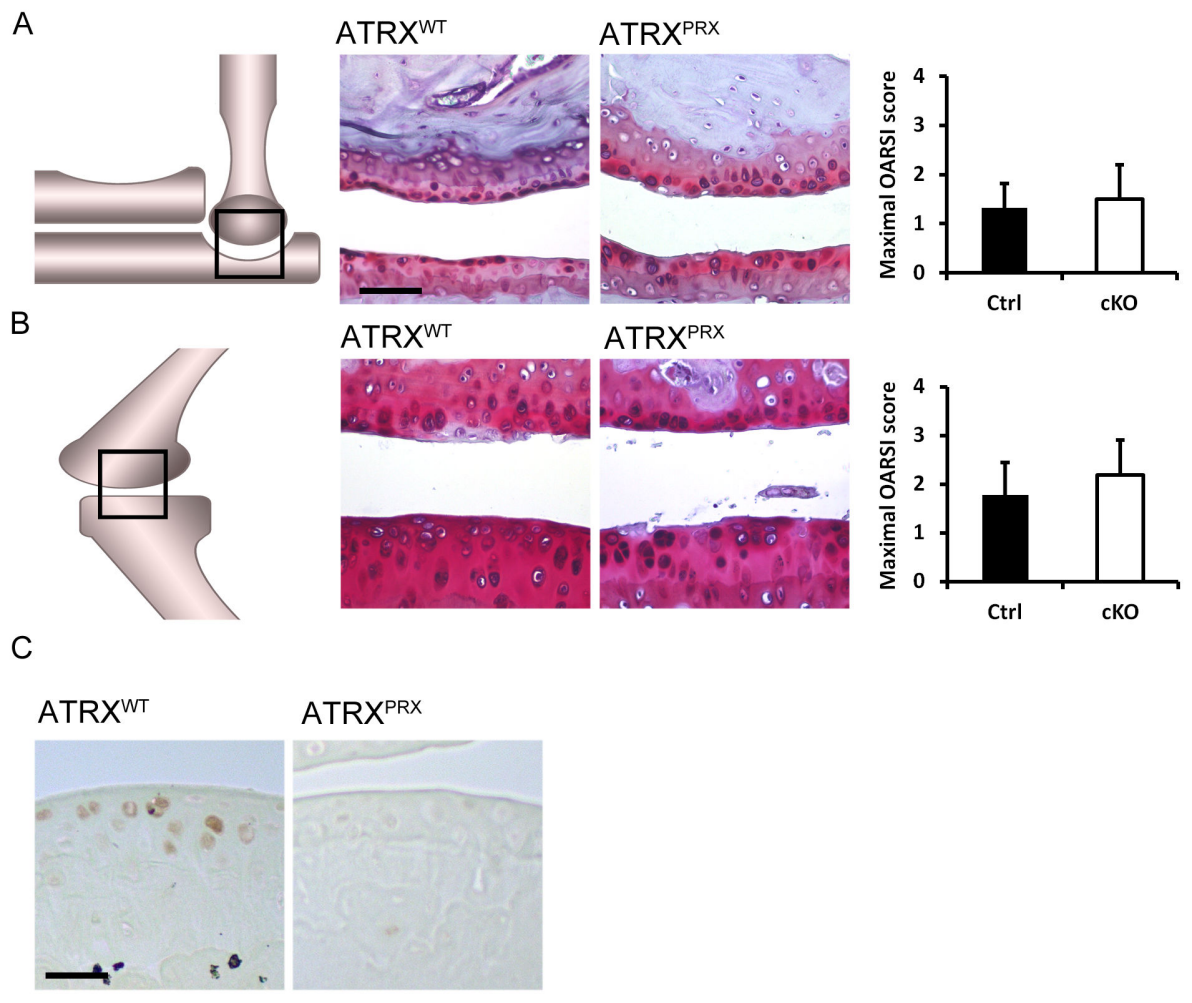
Characterisation of joint osteoarthritis in *Atrx* ^*Prx1*^ mice. A) Mild fibrillation and fissuring of the articular surfaces was observed in elbows of control or mutant mice, and no difference was seen between genotypes. Scale bar = 96 µm. B) Integrity of the knee was similar in control and mutant mice. C) Immunohistological staining of articular cartilage from the elbows of mice, demonstrating that ATRX protein is present in normal aged articular cartilage, but absent from the nuclei of chondrocytes derived from ATRX deficient forelimb mesenchyme. Scale bar = 48 µm.

*Atrx* ^*Prx1*^ mice were previously characterised and display brachydactyly and abnormal gait[27]. We now examined the joints to determine if structural variations in the limb might alter kinematics and thus lead to joint disease. Forelimb and hindlimb joints from *Atrx* ^*Prx1*^ mice were examined for osteoarthritis at two years of age (Figure 2). Although the forelimbs of *Atrx* ^*Prx1*^ are derived from ATRX-depleted mesenchyme, no difference was seen in the articular cartilage of the forelimb joint (Figure 2A). Control animals had a mean score of 1.32 and mutants has a mean score of 1.50 (p= 0.82). Similar to other models of ATRX loss in the skeleton, *Atrx* ^Prx1^ mice do not demonstrate knee osteoarthritis, with a maximal OA score of 2.18 in mutant knees and 1.78 in control littermates (p=0.69) (Figure 2B). Loss of ATRX in articular cartilage derived from ATRX-deficient forelimb mesenchyme was confirmed by staining with IHC (Figure 2C).

### *Atrx* ^*Col1*^ mice exhibit reduced growth without growth plate abnormalities

**Figure 3:**
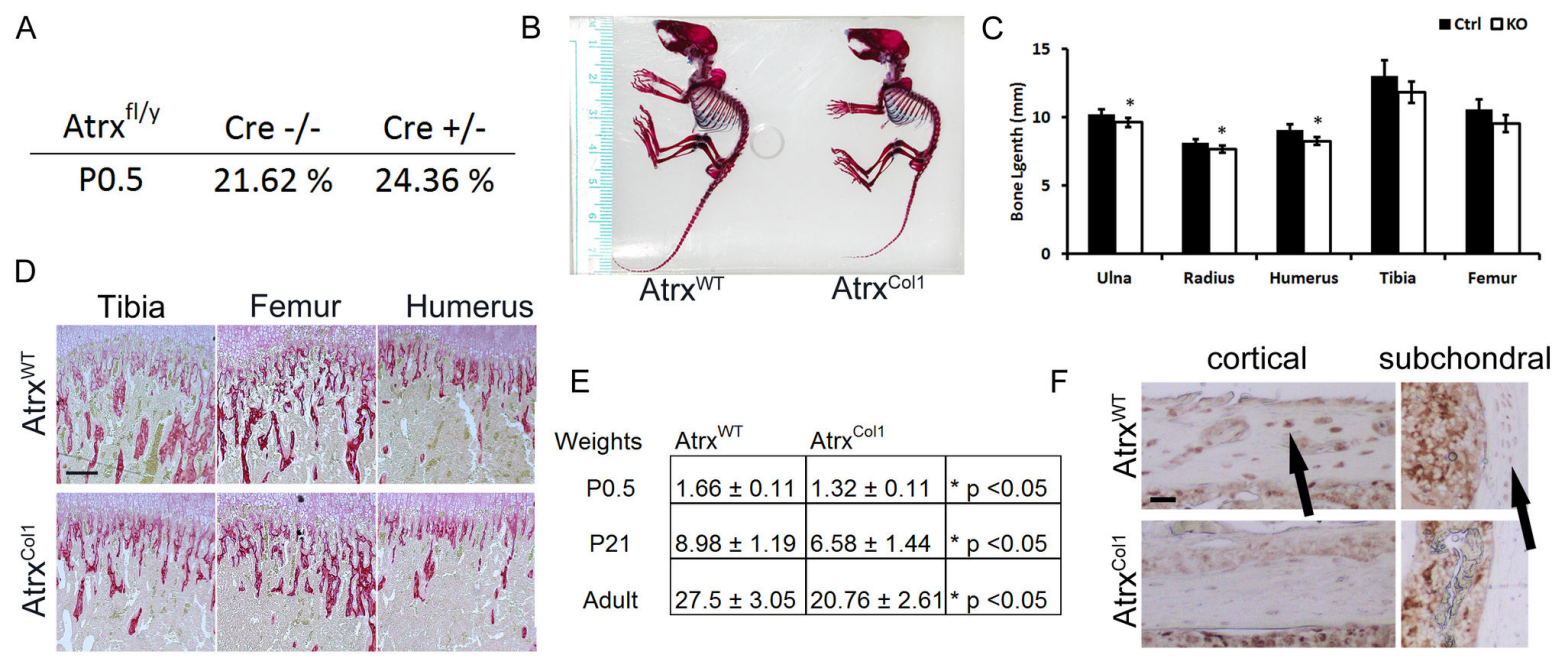
*Atrx* ^*Col1*^ mice exhibit reduced growth without growth plate abnormalities. A) Mice lacking Atrx in osteoblasts are born at normal Mendelian ratios. B) Overall skeletal morphology of *Atrx* ^*Col1*^ examined by alican blue/alizarin red stain reveals reduced overall size and shortened limbs. C) Longitudinal measurements of the bones of the axial skeleton demonstrate that all bones in the mutant are shortened, and forelimb bones show significant shortening compared to controls N=5 (* p<0.05). D) Mineralised trabecular bone area below the growth plate at weaning is not different between genotypes upon visualization of sections stained with picrosirius red staining. Scale bar = 192 µm. E) *Atrx* ^*Col1*^ mice are slightly smaller by body weight compared to littermates at birth, weaning and adulthood. F) IHC staining for Atrx protein demonstrates reduced Atrx in Atrx^Col1^ cortical and subchondral bone. Scale bar = 48 µm.

To examine the role of ATRX in bone-forming osteoblasts, the Col1a1-Cre driver line was utilized[26]. *Atrx* ^*Col1*^ mice were born at normal Mendelian ratios, showing that loss of *Atrx* in the osteoblast does not lead to embryonic or perinatal lethality (Figure 3A). In the neonatal period *Atrx* ^*Col1*^ mice exhibited reduced growth compared to their control littermates, and at weaning they were smaller and had shorter limbs (Figure 3B). Bone length measurements of the appendicular skeleton showed that the forelimb long bones of *Atrx* ^*Col1*^ mice (ulna, radius, humerus) are significantly shorter than those of controls, while the shortening of femur and tibia was not statistically significant (Figure 3C). Histological analysis of long bones by picrosirius red staining did not show any changes in growth plate morphology or trabecular density between genotypes (Figure 3D, and Figures S2,S3). Additionally, establishment of the secondary ossification center was not delayed in long bones of 10 day old mutant mice, as determined by safranin-O staining (not shown). At one year of age, *Atrx* ^*Col1*^ mice remained smaller than control littermates and did not catch up in weight (Figure 3E). Loss of ATRX in bone was confirmed by IHC staining with rabbit anti-ATRX (Prestige Antibodies, Sigma Aldrich) in cortical and subchondral bones (Figure 3F).

### *Atrx* ^*Col1*^ mice have normal mineralisation and circulating IGF-1 

**Figure 4:**
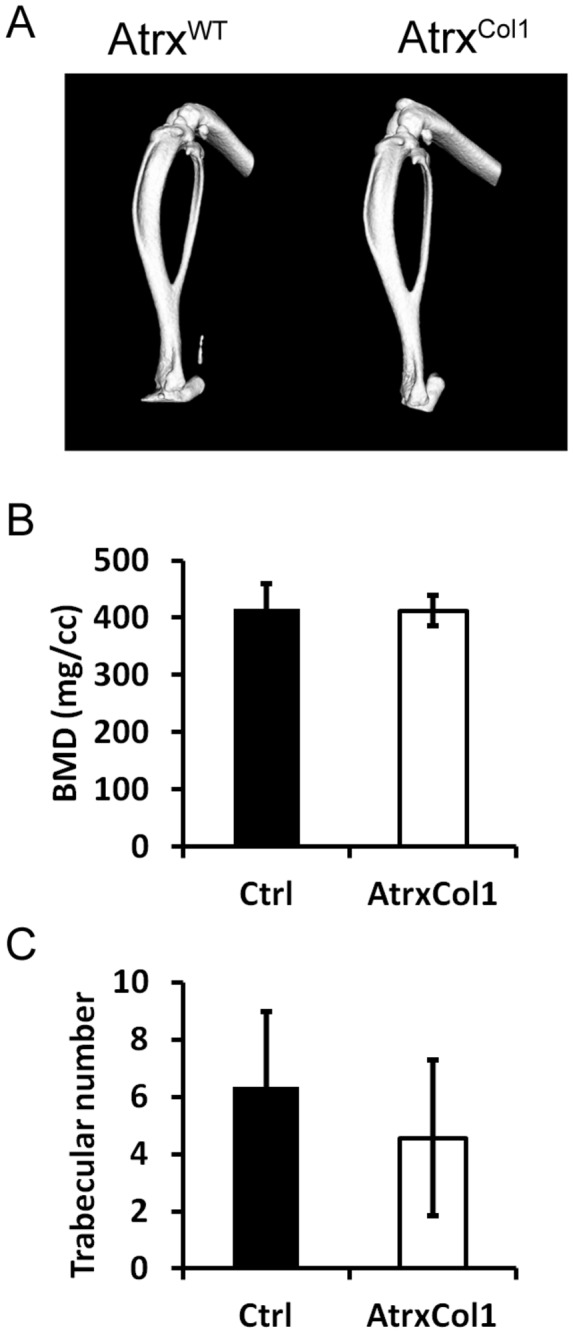
*Atrx* ^*Col1*^ mice have normal mineralisation. A) Representative tibias from weaned *Atrx* ^*Col1*^ showing that there was no gross morphological differences in the long bones between genotypes. B) No difference in bone mineral density (p = 0.90) was observed between *Atrx* ^*Col1*^ mice and controls. Error Bars - SD, N=3 C) Trabecular number was unchanged between *Atrx* ^*Col1*^ mice compared with controls (p = 0.46). Micro-CT data were obtained from hind legs using MicroView 3D software Error Bars - SD, N =3.

Micro-computed tomography (Micro-CT) analysis was performed at weaning and showed no changes in endochondral or intramembranous ossifications. Tibias appeared to have no gross morphological differences (Figure 4A) and trabecular bone mineral density (BMD) was unchanged between genotypes (Figure 4B) (N = 3, p >0.05). Trabecular number was also not significantly changed when analysed by Micro-CT (Figure 4C). 

 IGF-1 promotes longitudinal bone growth and reduced IGF-1 is associated with limb shortening[40]. To determine a possible endocrine component to the dwarfism in *Atrx* ^*Col1*^ mice, levels of circulating IGF-1 were measured by ELISA. This analysis revealed no significant difference in IGF-1 levels between control and mutant mice (207.433 ± 35.4 ng/ml vs. KO: 252.010 ± 56.8 ng/ml, respectively p =0.17).

**Figure 5:**
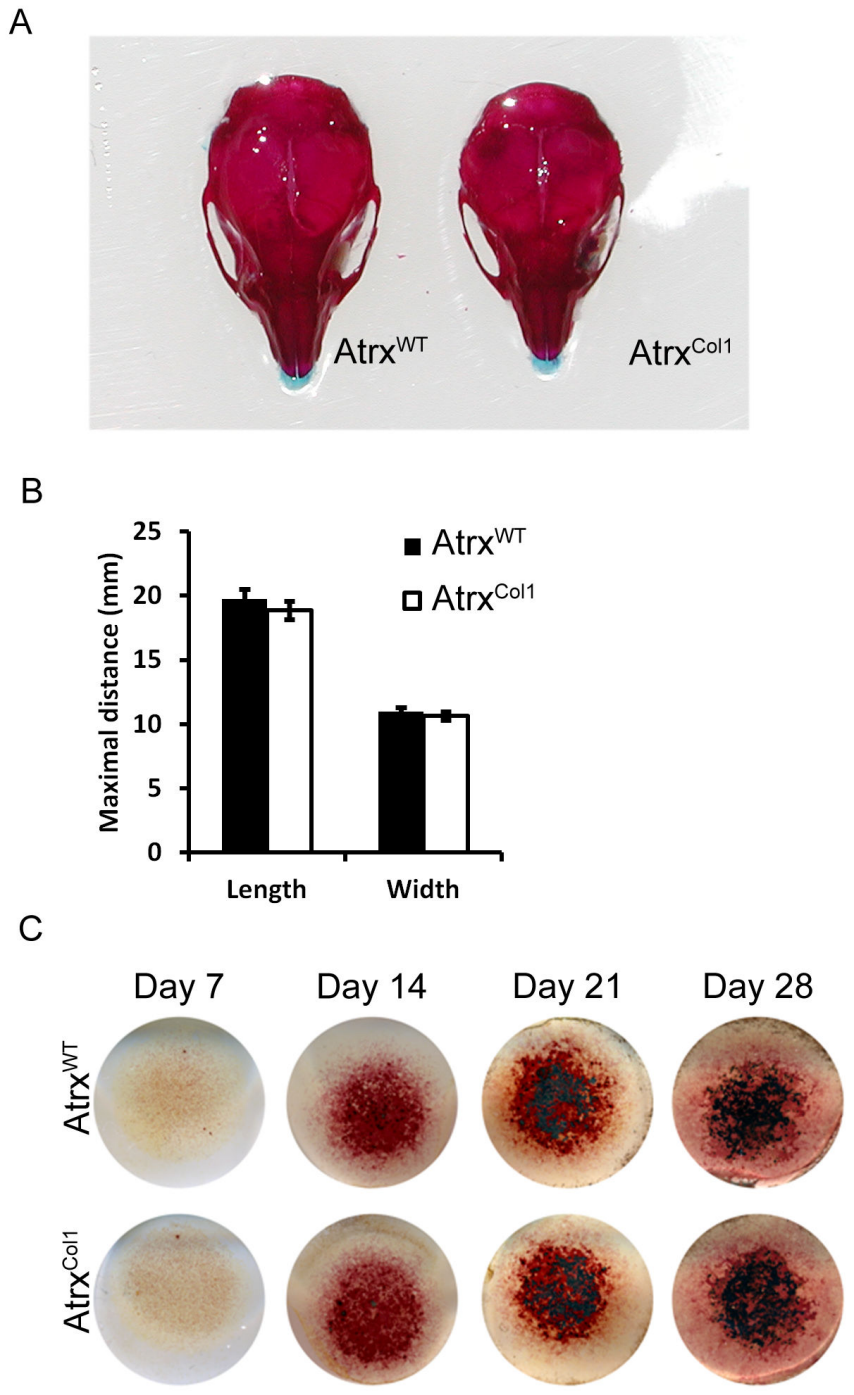
Ossification is not impeded or delayed by Atrx depletion in osteoblasts. A) Ossification of the skull is normal in *Atrx* ^*Col1*^ mice, and no changes are seen in suture formation. B) Skull length and width are not significantly affected in *Atrx* ^*Col1*^ mice compared to controls. N=5, Error bars - SD C) Mineralisation of high-density osteoblast cultures. Control and Atrx-null osteoblasts show mineralised nodules (black) after two weeks in osteogenic media, and mineralise at similar rates at three and four weeks. Representative images from five trials.

 To further investigate the role of ATRX in osteogenesis, we compared mineralisation in calvarial cells from control and *Atrx^Col1^* mice. Unlike long bones, which form through a cartilage intermediate, calvarial bone forms by intramembranous ossification and does not contain cartilage. Examination of skeletal preparations from 21-day-old mutant mice revealed no differences in calvarial structure or suture formation (Figure 5A). Mutant skulls appeared smaller than those of littermates, but were not significantly smaller by length (p=0.15) or width (p=0.14) (Figure 5B). To analyze mineralisation by ATRX-depleted osteoblasts, calvarial cells in 3D micromass culture were examined. Osteogenic media was supplemented with β-glycerophosphate and ascorbic acid for a four-week time course. The extent of differentiation and formation of mineralised nodules was assayed by alkaline phosphatase and von Kossa staining. While cultures increased mineralization over the time course as expected, no difference was observed between genotypes at any time over the four-week culture (Figure 5C). 

### Loss of ATRX in bone does not lead to osteoarthritis

**Figure 6:**
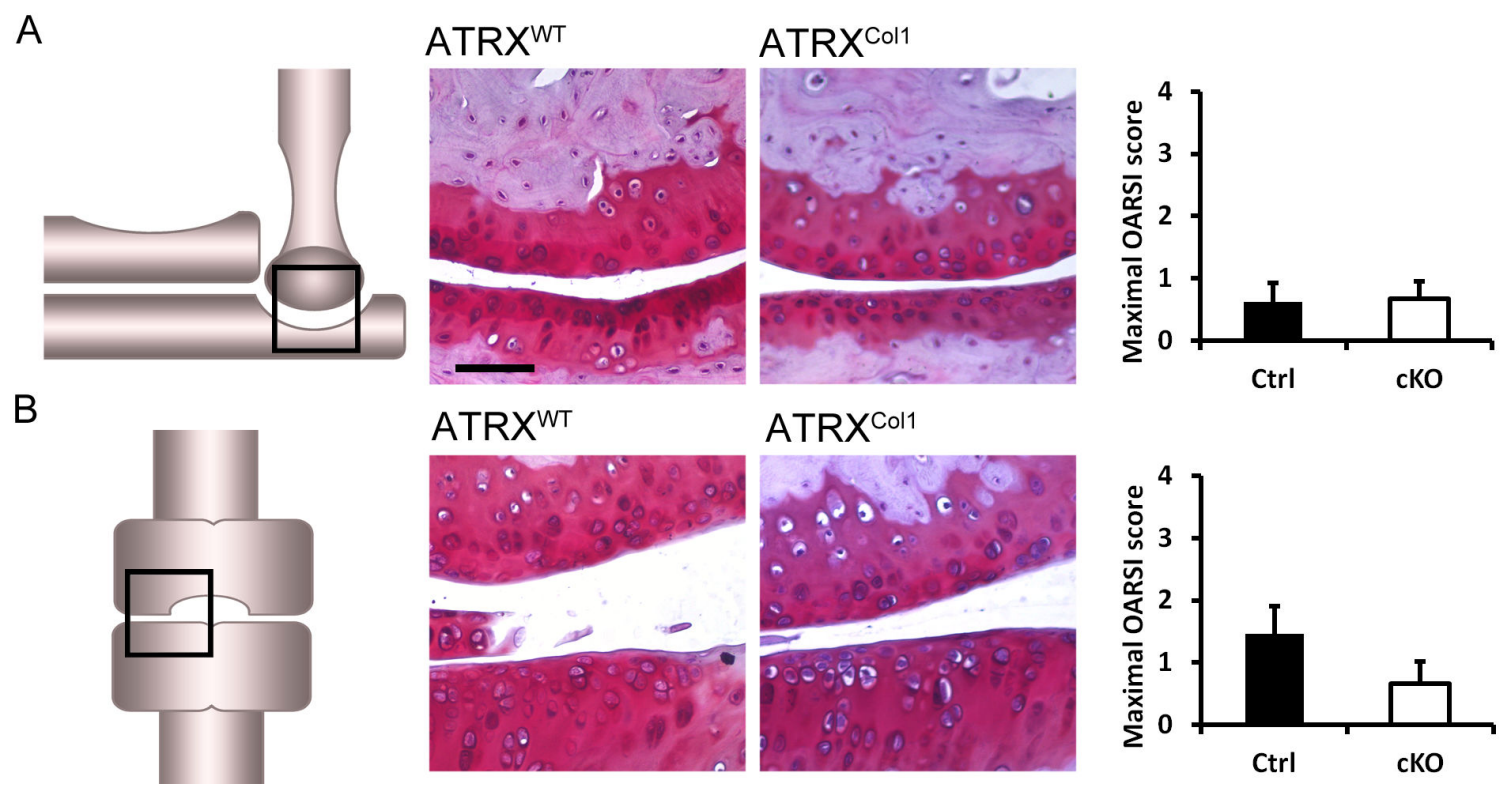
Characterisation of joint osteoarthritis in *Atrx* ^*Col1*^ mice. A) Fibrillation or fissuring of the articular surfaces was not observed in elbows of control or mutant mice, demonstrating very mild OA in both genotypes. Scale bar = 96 µm. B) Joint integrity of the medial surface of the knee was similar in control and mutant mice. N = 4, Error bars = SD.

 To determine if loss of ATRX in osteoblasts would lead to joint damage and osteoarthritis, joints of *Atrx* ^*Col1*^ mice were examined for osteoarthritis at one year of age (Figure 6). *Atrx* ^*Col1*^ mice expressed the same amount of ATRX in articular cartilage as control littermates, as expected with this Cre driver line (data not shown). Maximal OARSI scores of osteoarthritis in the elbows of Atrx^Col1^ mice were low in both genotypes; control animals had a mean score of 0.63 and mutants has a mean score of 0.67 (p= 0.92) (Figure 6A). Knee osteoarthritis scores were slightly higher, with control animals having an averaged maximal score of 1.46 for any quadrant of frontal knee sections and ATRX-deficient animals having a score of 0.67 (Figure 6B). These values were not significantly different (p=0.20). 

## Discussion

 Loss-of-function mutations in the human *ATRX* gene are associated with skeletal defects and high incidence of dwarfism. Mouse models of ATR-X syndrome have similar phenotypes, and demonstrate premature aging[25,27]. We report that aged mice lacking Atrx in skeletal tissues (*Atrx* ^*Col2*^, *Atrx* ^*Col1*^, and *Atrx* ^*Prx1*^) show very limited skeletal defects, with no associated increase in osteoarthritis. 

*Atrx* ^*Col2*^ mice exhibit minimal skeletal defects during development[24], so it was not completely surprising that cartilage integrity was maintained during normal aging. Adult *Atrx* ^*Col2*^ mice displayed minor fibrillation and loss of proteoglycan in the articular cartilage, but at equal levels as aged-matched control littermates. Thus, given the relative stability of Atrx-null articular cartilage, Atrx does not appear to be required for structural maintenance of articular cartilage. 

No phenotype was observed in the *Atrx* ^*Prx1*^ mouse, despite biomechanical differences observed between genotypes leading to stride alterations[27]. *Atrx* ^*Prx1*^ mice have shortened digits and reduced function, but only demonstrate Atrx loss in the forelimb and not the hindlimb. Therefore, it was expected that no differences would be observed in the hindlimb articular cartilage, as the hindlimbs of these animals can be considered wild-type due to the expression of the forelimb cre during development[28].

While this study is reflective of the majority of OA cases related to aging, it does not consider the role of injury in disease progression. Injury can also be underlying cause of OA, and further studies challenging the animals with joint trauma could be considered in the future. 

In the present study, we found that loss of Atrx in osteoblasts causes mild but significant shortening of the limbs. Given the similar, but more severe, dwarfism observed when *Atrx* is deleted in the developing forebrain and anterior pituitary [25], it is possible that this dwarfism is due to an indirect effect, because of unspecific Cre expression. The possibility of an indirect effect is supported by the fact that ex-vivo cultures of ATRX-null osteoblasts mineralise normally. Evidence suggests that the Col1a1-cre used in this study is capable of some LoxP recombination in the brain, so there remains a possibility that part of the dwarfism phenotype may be due to leaky *Atrx* recombination in the brain[41]. On the other hand, normal circulating IGF1 levels suggest that reduced skeletal growth is due to disruption of a bone-intrinsic role of Atrx.

 In previous reports, a direct or indirect role for ATRX in the development of the skeleton has been characterised[24,25,27]. Alterations in the expression of other chromatin-remodelling proteins in the skeleton can lead to severe malformations[42,43]. For example, dominant negative mutations in the SWI/SNF subunit *BRG1* reduce levels of the transcription factor RUNX2, an important regulator of osteogenesis[43]. However, no equivalent changes were observed in the expression of *Runx2* or other osteogenic genes in Atrx-null calvaria (data not shown). Osteoblast precursors isolated from *Atrx* ^Col1^ mice matured in high density micromass culture and formed mineral at the same rate as cells from control littermates. Finally, trabecular density was unaffected when examined histologically, as well as by Micro-CT analysis. Therefore, we conclude that loss of Atrx in osteoblasts does not impede their ability to mineralise tissue. 

 When Atrx is lost in the developing limb bud, DNA damage is observed as an accumulation of y-H2AX-positive nuclei in forelimb cells[27]. This single focus co-localises with DNA damage response factor 53BP1 at the nuclear lamina, and forelimbs lacking Atrx have higher incidence of TUNEL staining[27]. However, loss of Atrx in this model caused only a slight increase in apoptotic cells compared to other tissues such as brain, trophoblast, muscle and Sertoli cells[20,21,23,44]. Thus, our findings suggest that the protective effect of ATRX against DNA damage and replicative stress is tissue-specific, and that skeletal tissues are more resistant to the effects of Atrx loss. 

 In conclusion, ATRX deletion in various skeletal lineages confers only mild, tissue-specific defects and does not fully recapitulate the skeletal phenotypes of ATR-X syndrome. Furthermore, there is no increased incidence of joint osteoarthritis and no difference in the progression of skeletal pathologies in adulthood. Although *ATRX* mutations are associated with skeletal defects in humans, the underlying cause is likely indirect due to reduced function of ATRX in other tissues[25]. In conclusion, ATRX is important in the developing skeleton prior to chondrocyte differentiation and during mineralisation, but is not important for maintenance of adult skeletal tissues. 

## Supporting Information

Figure S1 Figure S1A. MMP13 expression is similar in the articular cartilage of *Atrx* ^*Col2*^ mice compared to controls. Representational immunohistochemical stains of articular cartilage in *Atrx* mutant and control mice performed on paraffin sections. Scale bar = 200 μm. **Figure S1B**. MMP13 expression was not significantly changed in *Atrx* ^*Col2*^ mice. Analysis was performed by counting the percentage of MMP13-positive cells in paraffin sections using representational subsets of tibial articular cartilage. The average percentage of positive cells per articular surface was 35.7 ± 0.67% for controls and 39.3 ± 14.2% positive cells for mutants(n= 8). Control and knockout sections were not significantly different (p> 0.05). **Figure S1C**. Tibial aggrecan fragmentation is unchanged in Atrx^Col2^ mice. Representational stains for aggrecan fragments in control and knockout mice on 5μm paraffin sections. Scale bar = 200μm. **Figure S1D**. Tibial aggrecan fragmentation in unchanged in Atrx^Col2^ mice. The average number of aggrecan fragment-positive cells in controls was 34.02 ± 5.92% (Mean ± SEM) (n= 5). The mean percentage of positive cells in knockouts was 22.8 ± 10.65% (n= 8). Mean percentages of aggrecan fragment-positive cells between controls and knockout tibiae were not significantly different (p> 0.05). **Figure S1E**. The type II collagen -positive zone in articular cartilage of knees of control and Atrx^Col2^ mice. Immunohistochemistry stains on paraffin knee sections for collagen 2. Scale bar = 200μm. **Figure S1F**. The thickness of the type II collagen-positive zone in the articular cartilage of the tibia and femur is not different in *Atrx* ^*Col2*^ mice. Three measurements of the type II collagen -positive zone in the tibia and femur were taken. The average tibial thickness of the type II collagen positive zone in controls vs. knockout mice was 87.993 ± 8.827 μm vs. 90.065 ± 5.529 μm (Mean ± SEM) (n= 5). The average femoral thickness of the type II collagen-positive zone in controls and knockout mice was 82.080 ± 8.820 μm vs. 81.142 ± 5.084 μm (n= 8). Neither the tibiae nor femurs of control and knockout mice showed any significant differences (p> 0.05).
(TIF)

Figure S2 **Growth plate measurements in *Atrx*^*Col1*^ mice.** No significant difference was seen in the length of the resting, proliferating or hypertrophic zones in long bones in *Atrx* ^*Col1*^ or Control littermates at weaning (N = 3 littermate pairs; two-tailed T-test). Error bars - SD.
(TIF)

Figure S3 **Trabecular area quantification in *Atrx*^*Col1*^ mice.** Quantification of the area of mineralised trabecular area shows that mineralisation is unaffected in *Atrx* ^*Col1*^ mice. No difference in trabecular area below the growth plates in the tibia, femur or humerus between *Atrx* ^*Col1*^ mice and controls.
(TIF)
