# Supplementary figures and images for: Loss of ATRX Does Not Confer Susceptibility to Osteoarthritis
Source: PLoS One. 2013 Dec 30;8(12):e85526. doi: 10.1371/journal.pone.0085526 (PMC3875582; doi:10.1371/journal.pone.0085526)

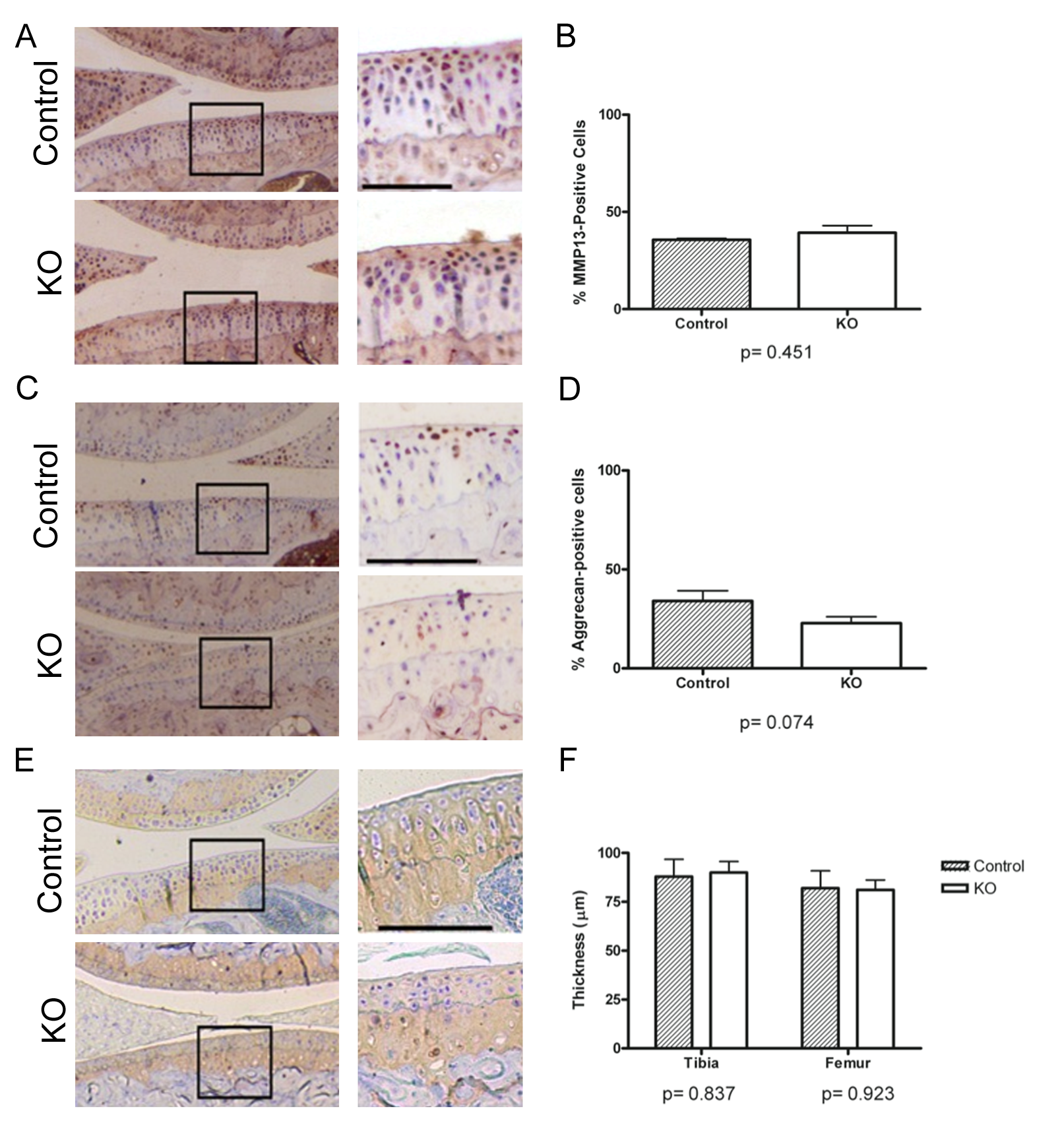

Supplement: Figure S1 — A. MMP13 expression is similar in the articular cartilage of Atrx Col2 mice compared to controls. Representational immunohistochemical stains of articular cartilage in Atrx mutant and control mice performed on paraffin sections. Scale bar = 200 μm. Figure S1B. MMP13 expression was not significantly changed in Atrx Col2 mice. Analysis was performed by counting the percentage of MMP13-positive cells in paraffin sections using representational subsets of tibial articular cartilage. The average percentage of positive cells per articular surface was 35.7 ± 0.67% for controls and 39.3 ± 14.2% positive cells for mutants(n= 8). Control and knockout sections were not significantly different (p> 0.05). Figure S1C. Tibial aggrecan fragmentation is unchanged in AtrxCol2 mice. Representational stains for aggrecan fragments in control and knockout mice on 5μm paraffin sections. Scale bar = 200μm. Figure S1D. Tibial aggrecan fragmentation in unchanged in AtrxCol2 mice. The average number of aggrecan fragment-positive cells in controls was 34.02 ± 5.92% (Mean ± SEM) (n= 5). The mean percentage of positive cells in knockouts was 22.8 ± 10.65% (n= 8). Mean percentages of aggrecan fragment-positive cells between controls and knockout tibiae were not significantly different (p> 0.05). Figure S1E. The type II collagen -positive zone in articular cartilage of knees of control and AtrxCol2 mice. Immunohistochemistry stains on paraffin knee sections for collagen 2. Scale bar = 200μm. Figure S1F. The thickness of the type II collagen-positive zone in the articular cartilage of the tibia and femur is not different in Atrx Col2 mice. Three measurements of the type II collagen -positive zone in the tibia and femur were taken. The average tibial thickness of the type II collagen positive zone in controls vs. knockout mice was 87.993 ± 8.827 μm vs. 90.065 ± 5.529 μm (Mean ± SEM) (n= 5). The average femoral thickness of the type II collagen-positive zone in controls and knockout mice was [file pone.0085526.s001.tif]

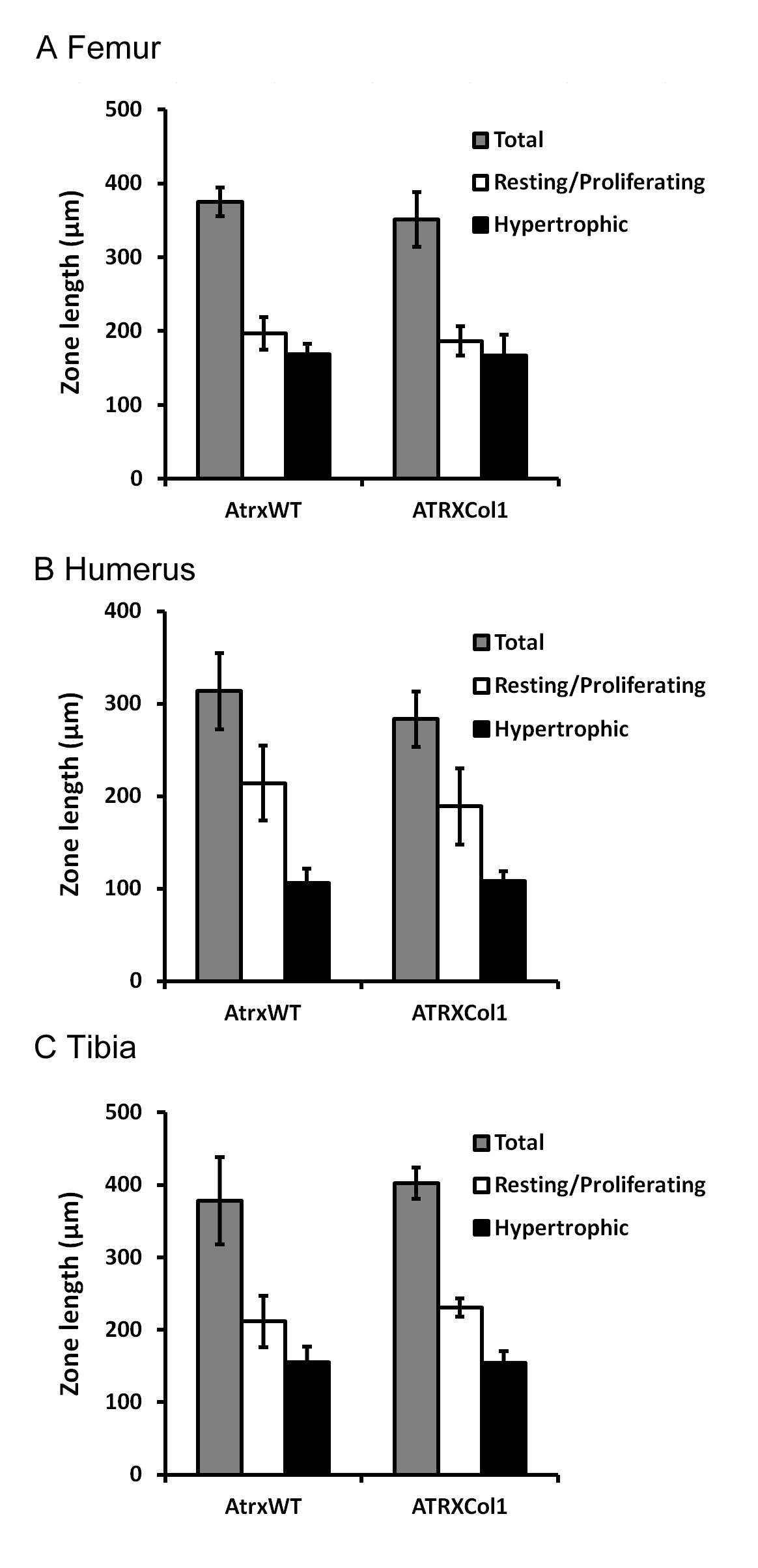

Supplement: Figure S2 — Growth plate measurements in AtrxCol1 mice. No significant difference was seen in the length of the resting, proliferating or hypertrophic zones in long bones in Atrx Col1 or Control littermates at weaning (N = 3 littermate pairs; two-tailed T-test). Error bars - SD. (TIF) [file pone.0085526.s002.tif]

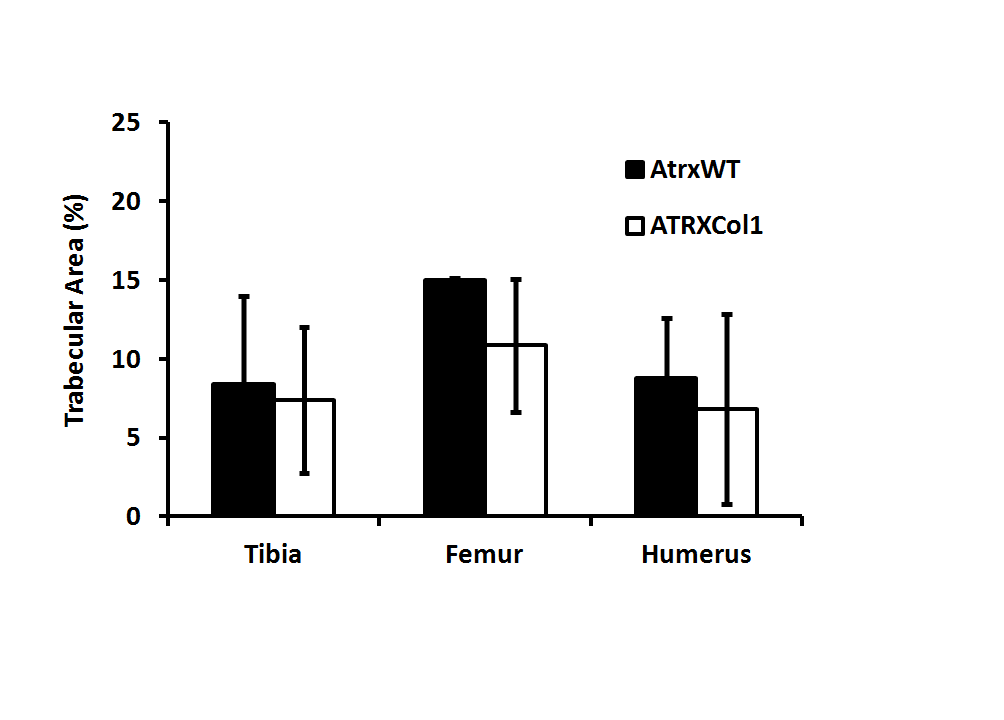

Supplement: Figure S3 — Trabecular area quantification in AtrxCol1 mice. Quantification of the area of mineralised trabecular area shows that mineralisation is unaffected in Atrx Col1 mice. No difference in trabecular area below the growth plates in the tibia, femur or humerus between Atrx Col1 mice and controls. (TIF) [file pone.0085526.s003.tif]
